# Establishment of Murine Gammaherpesvirus Latency in B Cells Is Not a Stochastic Event

**DOI:** 10.1371/journal.ppat.1004269

**Published:** 2014-07-31

**Authors:** Jérémie Decalf, Cristina Godinho-Silva, Diana Fontinha, Sofia Marques, J. Pedro Simas

**Affiliations:** Instituto de Medicina Molecular, Faculdade de Medicina, Universidade de Lisboa, Lisboa, Portugal; University of Southern California, United States of America

## Abstract

Murid γ-herpesvirus-4 (MuHV-4) promotes polyclonal B cell activation and establishes latency in memory B cells via unclear mechanisms. We aimed at exploring whether B cell receptor specificity plays a role in B cell susceptibility to viral latency and how this is related to B cell activation. We first observed that MuHV-4-specific B cells represent a minority of the latent population, and to better understand the influence of the virus on non-MuHV-4 specific B cells we used the SW_HEL_ mouse model, which produce hen egg lysozyme (HEL)-specific B cells. By tracking HEL^+^ and HEL^−^ B cells, we showed that in vivo latency was restricted to HEL^−^ B cells while the two populations were equally sensitive to the virus in vitro. Moreover, MuHV-4 induced two waves of B cell activation. While the first wave was characterized by a general B cell activation, as shown by HEL^+^ and HEL^−^ B cells expansion and upregulation of CD69 expression, the second wave was restricted to the HEL^−^ population, which acquired germinal center (GC) and plasma cell phenotypes. Antigenic stimulation of HEL^+^ B cells led to the development of HEL^+^ GC B cells where latent infection remained undetectable, indicating that MuHV-4 does not benefit from acute B cell responses to establish latency in non-virus specific B cells but relies on other mechanisms of the humoral response. These data support a model in which the establishment of latency in B cells by γ-herpesviruses is not stochastic in terms of BCR specificity and is tightly linked to the formation of GCs.

## Author Summary

Murid γ-herpesvirus-4 (MuHV-4) is a good model to study infectious mononucleosis in mice, in which the virus ultimately establishes life-long latency in B cells. Whereas several viral proteins have been shown to modulate B cell behavior, in the present study we aimed at clarifying the parameters that dictate the establishment of viral latency from the B cell perspective. Indeed, the B cell repertoire is highly diverse and it remains unknown whether latency takes place randomly in B cells. To study this question, we isolated latently infected B cells in which we observed a low frequency of virus-specific B cells, suggesting that viral latency is not restricted to this population. To better understand MuHV-4 influence on non-virus specific B cells, we then followed the fate of B cells specific for a foreign antigen, hen egg lysozyme (HEL). While in vitro experiments showed that HEL-specific B cells could be acutely infected by MuHV-4, these cells were resistant to MuHV-4 latent infection in vivo. These results suggest that while establishment of γ-herpesvirus latency is not restricted to virus-specific B cells, it does not take place randomly in B cells and relies on mechanisms that remain to be identified.

## Introduction

The murid γ-herpesvirus-4 (MuHV-4, also known as MHV-68 or γHV-68) has led to valuable insights in understanding human γ-herpesvirus related diseases caused by Epstein-Barr virus (EBV) and Kaposi's sarcoma associated herpesvirus (KSHV) [1]. Whereas primo infection by γ-herpesviruses can be responsible for lymphoproliferative disorders in immune competent hosts, they are usually well controlled [2]. As with EBV, MuHV-4 is mainly lymphotropic and establishes latency in class-switched and germinal center (GC) B cells [3], [4]. The course of the infection in mice is now well described (see [5] and [1]). Upon intranasal inoculation, infection starts with an acute lung infection controlled by the CD8^+^ T cell response. The virus then disseminates to secondary lymph organs via serial events of lymphoid/myeloid cellular exchanges [6] where it promotes a CD4-dependent polyclonal B cell response and finally establishes latency in long-lived memory B cells [1], [5], [7], [8]. This polyclonal B cell activation can lead to the emergence of auto-antibodies but MuHV-4 infection is usually not associated with the development of auto-immune diseases or lymphomas in immune competent mice [9]. CD4^+^ T cells, and in particular follicular helper T cells [10], have been shown to be essential for the establishment of MuHV-4 latency. Antibody-mediated depletion experiments [11], [12] as well as work performed on MHC class II deficient mice [13] (which are CD4^+^ T cells deficient) have led to similar observations, that the absence of CD4^+^ T cells leads to lower latency levels.

On the virus side, few proteins have been shown to be involved in the establishment of latency [1]. Among them, M2 has received particular interest for its ability to interfere with B cell activation. Studies performed with M2-deficient MuHV-4 have shown its essential role in the establishment of latency, although it is not required for acute lung infection [14], [15]. Biochemical analysis have established that M2 is able to interact with the Fyn/Vav, Plcγ2 and PI3K pathways, involved in BCR signaling [16]–[18]. In vivo, B cells infected by M2-deficient MuHV-4 have been shown to acquire a GC phenotype comparable with the WT virus, but were unable to class-switch and differentiate into plasma cells [19]. MuHV-4 LANA has recently been shown to stabilize cellular Myc and promotes its activity, leading to B cell proliferation, a process required for GC formation and viral latency [20].

The lower level of viral latency observed in mice deprived of CD4^+^ T cells as well as with M2-deficient MuHV-4 are good examples showing that the establishment of MuHV-4 latency relies on mechanisms that mix the physiologic B cell response and the intervention of viral modulators. Several questions remain to be explored to better understand this complex interaction: How does MuHV-4 trigger a polyclonal B cell activation? Are latently infected B cells also polyclonal, or is latency restricted to MuHV-4 specific B cells? Finally, what are the respective roles for the virus and the B cells in the establishment of latency?

Until recently, those were difficult questions to address experimentally because of two major hurdles on the virus and the B cell sides. At peak of latency (∼14 days post-infection in C57BL/6 mice), latently infected B cells represent a low percentage of total B cells [3], [4] and tracking these cells was impossible until the development of a YFP expressing MuHV-4 [4]. On the B cell side, questions concerning BCR specificity are delicate to address due to the enormous diversity of the B cell repertoire and to the difficulty to trace one clonal population, but major improvement was made with the development of the switch hen egg lysozyme mice (SW_HEL_) [21]–[24]. Based on the MD4 model [25], SW_HEL_ mice have been engineered to contain up to ∼10% of HEL-specific B cells. But contrary to MD4 mice, SW_HEL_ HEL^+^ B cells can perform GC reactions in a competitive environment, class-switch and differentiate in long-lived memory B cells. Moreover, HEL-specific (HEL^+^) and non-specific (HEL^−^) populations can easily be distinguished by direct staining of the BCR with fluorescently labeled HEL.

In the present study, we aimed at clarifying the role of BCR specificity in the establishment of MuHV-4 latency in B cells. Taking advantage of the SW_HEL_ mice and the YFP-MuHV-4 we designed experiments to study in parallel how MuHV-4 influences a normal B cell repertoire (HEL^−^) and a clonal population of non-virus specific B cells (HEL^+^) in order to determine in which population latency is established and how this relates with B cell activation.

## Results

### MuHV-4 latency is not restricted to virus-specific B cells

**Figure 1:**
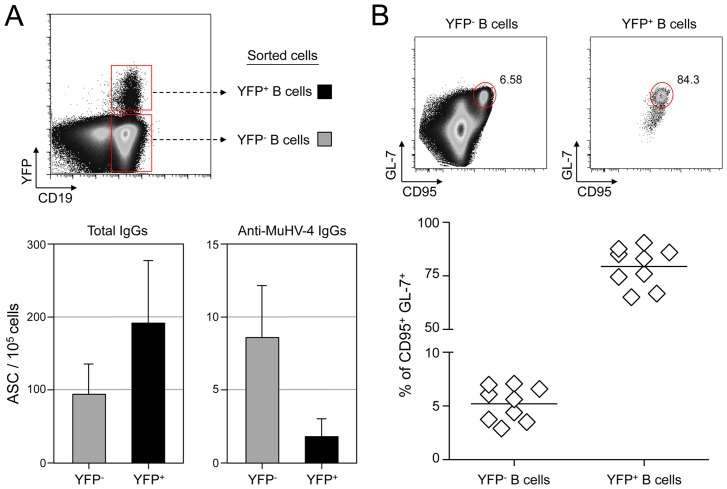
MuHV-4 specific B cells represent a minor part of infected and non-infected cells. (A) As presented in the FACS plot, infected (CD19^+^ YFP^+^, black bars) and non-infected (CD19^+^ YFP^−^, grey bars) B cells were sorted from C57BL/6 mice 14 dpi. Cells were used to assess the frequency of total (left) and MuHV-4 specific (right) antibody-secreting cells (ASC) by ELISPOT. These data are from 3 independent experiments, in each experiment spleens from 3 to 4 mice where pooled before performing the sorts. Post-sorts purities were systematically >95% for YFP^+^ B cells and >98% for YFP^−^ B cells. (B) To monitor the frequency of GC cells in YFP^−^ and YFP^+^ B cells, spleens from YFP-MuHV-4 infected C57BL/6 mice (n = 9) were isolated at 14 dpi for FACS analysis. All the mice harbored a frequency of infected B cells >0,05%, with at least 500 events in the YFP^+^ gate. A representative FACS plot showing the frequency of CD95^+^ GL-7^+^ cells is shown on top and compiled data obtained from 4 independent experiments is shown below. Bars represent the average percentage.

To evaluate the frequency of MuHV-4 specific B cells in infected and non-infected populations, we challenged C57BL/6 mice with YFP-MuHV-4. Infected and non-infected CD19^+^ B cells were sorted at 14 dpi based on their YFP expression and used in an ELISPOT assay to evaluate the number of total IgGs and anti-MuHV-4 IgGs secreting cells (Figure 1A). Both YFP^−^ and YFP^+^ B cells showed a low frequency of virus-specific antibody-secreting cells (ASC) cells when compared to the total ASC. That is, ∼10% of total ASC for the YFP^−^, and ∼1% for the YFP^+^ populations, showing that in both populations the majority of ASC are not MuHV-4 specific. As the frequency of GC B cells (GL-7^+^, CD95^+^) is significantly different between YFP^+^ and YFP^−^ B cells (Figure 1B), we tried to refine our analysis by sorting infected and non-infected GC cells based on GL-7 and CD95 expression. Yet, purified cells died quickly and could not be used for ELISPOT assays, probably due to anti-CD95 induced apoptosis [26]. Our ELISPOT assay did not include the monitoring of IgGs specific for non-structural proteins. However, it would be unlikely if they accounted for the 90 to 99% of the ASC not detected in our anti-MuHV-4 IgGs assay. These data indicate that latent infection is not restricted to MuHV-4 specific B cells and that the virus is able to promote the activation of non-virus-specific B cells independently of their infection status. In order to explore these two points, we next used the SW_HEL_ mice [21], which allowed us to monitor MuHV-4 influence on non-virus-specific HEL^+^ B cells.

### HEL^+^ B cells are not latently infected by MuHV-4

**Figure 2:**
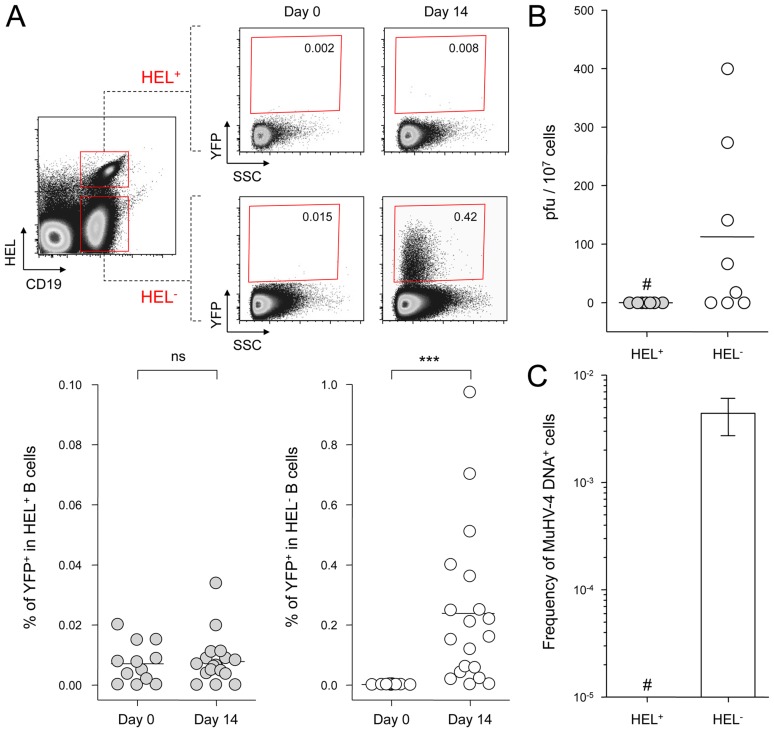
MuHV-4 latent infection is restricted to polyclonal HEL^−^ B cells. SW_HEL_ mice were left untreated or infected for 14 days with YFP-MuHV-4 virus at 10^4^ PFU. Splenocytes were isolated and analyzed by FACS or sorted for plaque assay and limiting dilution PCR analysis. HEL^+^ and HEL^−^ B cells were identified based on HEL-A647 binding and CD19 expression. (A) On top are representative FACS plots showing the percentage of YFP^+^ cells in both populations at 0 and 14 dpi. Data are compiled in the graphics below showing percentage of YFP^+^ cells in HEL^+^ (grey circles) and HEL^−^ (white circles) B cells at day 0 (n = 12) and 14 (n = 16) dpi. The bar represents the average percentage. (B) To detect presence of latent MuHV-4, a plaque assay was performed on sorted HEL^+^ and HEL^−^ B cells 14 dpi (n = 8). # indicates that no plaques were detected in HEL^+^ B cells and based on the average number of HEL+ B cells plated, we estimate the level of latent infection to be <1 PFU/382000 in this population. The analysis of lysed cells showed an absence of preformed viral particles in both populations. (C) Limiting dilution PCR was performed to detect viral genome in sorted HEL^+^ and HEL^−^ B cells from day 14 infected SW_HEL_ mice. Data are representative of two independent experiments in which splenocytes were pooled before sorting (n = 3 & n = 2). The frequency of cells harboring MuHV-4 DNA is shown, # indicates signal below the assay's limit of detection. We evaluated the frequency of MuHV-4 DNA^+^ cells in HEL^+^ B cells to be ≤1/36770 and ≤1/102891 for the two experiments we performed. Error bars indicate the 95% confidence interval. Purity of sorted cells was systematically above 90%.

SW_HEL_ mice were infected with YFP-MuHV-4 and we monitored YFP expression in HEL^+^ and HEL^−^ B cells 14 dpi (Figure 2A). While we did not detect YFP expression in the HEL^+^ population, HEL^−^ B cells harbored a frequency of YFP^+^ cells comparable with what has been previously reported in WT B cells [4]. We confirmed that HEL^−^ B cells are solely latently infected by sorting HEL^+^ and HEL^−^ B cells on which we monitored reactivation of latent virus by ex vivo explant co-culture assay (Figure 2B) and the presence of viral DNA by limiting dilution PCR (Figure 2C). It is important to note that HEL^−^ B cells emerge from a spontaneous replacement of the Vh10 exon encoding for the HEL-specific heavy chain leading to the reconstitution of a polyclonal repertoire [21], minimizing the impact of genetic differences between these two populations. However, HEL^+^ B cells only belong to the B-2 lineage, while HEL^−^ differentiate into both B-1 and B-2 B cells [21]. It is unknown in which population of B cells MuHV-4 latency is established. To evaluate if the B-2 bias of the HEL^+^ B cells would account for their resistance to latent infection, we phenotyped latently infected cells in C57BL/6 mice (Figure S1). B-2 B cells represented the vast majority of YFP^+^ B cells, but a small fraction of latently infected cells was also detected in B-1a and B-1b B cells. The proportion of B-2, B-1a and B-1b in YFP^+^ and YFP^−^ B cells corresponded to what has been described for naïve animals, with the B-2 lineage being dominant in splenic B cells [27]. Overall, these data make it unlikely that the B-2 commitment of HEL^+^ B cells would explain their resistance to MuHV-4 latency.

**Figure 3:**
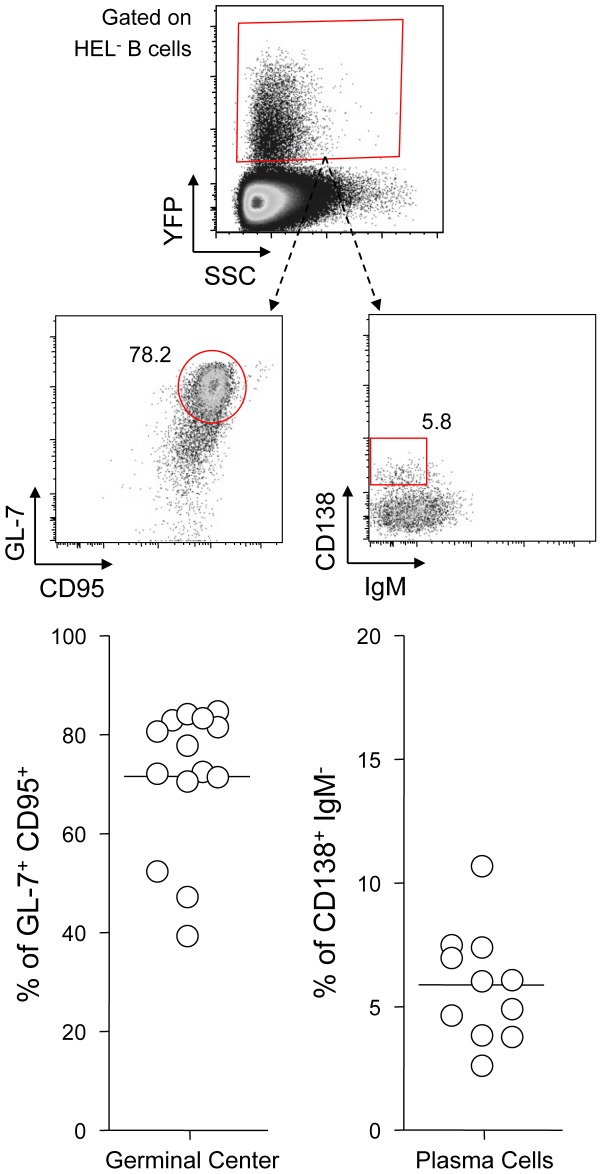
YFP^+^ HEL^−^ B cells differentiate into GC and plasma cells. Splenocytes were isolated from SW_HEL_ mice 14 dpi and analyzed by FACS. Cells were gated on the HEL^−^ CD19^+^ population. On top is shown a typical phenotype of YFP^+^ HEL^−^ B cells, monitoring GC (CD95^+^ GL-7^+^) and plasma cells (IgM^−^ CD138^+^). Data were compiled in the graphics below; showing the percentage of GC cells (n = 14) and plasma cells (n = 11) in YFP^+^ HEL^−^ B cells.

Finally, to monitor that MuHV-4 latency in SW_HEL_ HEL^−^ B cells reproduces what has been described in a normal repertoire, we determined the phenotype of YFP^+^ HEL^−^ B cells (Figure 3). As for WT B cells (Figure 1B), ∼75% of YFP^+^ HEL^−^ B cells harbored a GL-7^+^ CD95^+^ phenotype, with a minor fraction harboring a plasma cell phenotype and ongoing class-switch (CD138^+^ IgM^−^). Together these data show that HEL^+^ B cells are not latently infected by MuHV-4 while latency takes place normally in HEL^−^ B cells.

### HEL^+^ and HEL^−^ B cells are both sensitive to MuHV-4 infection in vitro

MuHV-4 poorly infects B cells in vitro [28], but work by Frederico et al overcame this hurdle by developing an in vitro co-culture assay, and showed that MuHV-4 transits by myeloid cells in order to get access to B cells [29]. Taking advantage of this experimental setting we investigated whether the absence of latently infected HEL^+^ B cells in vivo was due to an intrinsic resistance of these cells to the virus. As the YFP-MuHV-4 used for in vivo experiments allows the detection of latently infected cells we used in this experiment an EF1α-eGFP^+^ MuHV-4, in which GFP expression can be detected 48 h post infection. We used in parallel gp150^+^ and a gp150^−^ viruses, the later leading to a better B cell infection in co-culture assay [29].

**Figure 4:**
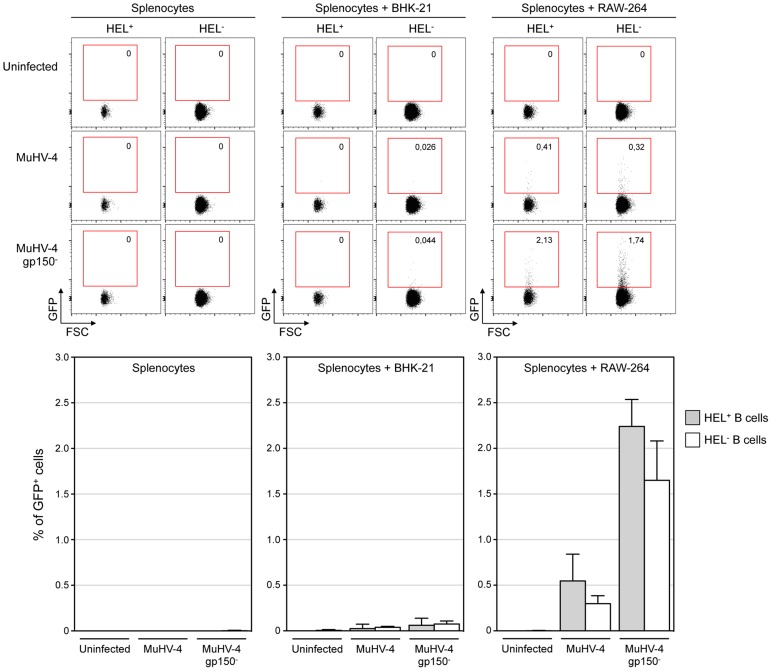
HEL^+^ and HEL^−^ B cells are both infected by MuHV-4 in vitro. In vitro infection by co-culture assay was performed as described in the material and methods. Briefly, freshly isolated SW_HEL_ splenocytes were exposed to free viruses or co-culture with BHK-21 or RAW-264 cells previously infected with EF1α-eGFP^+^ or EF1α-eGFP^+^-gp150^−^ MuHV-4. GFP expression in HEL^+^ and HEL^−^ B cells was monitored by FACS after 48 h of co-culture. Representative FACS plots are shown on top, compiled data representing average percentage and standard deviation are shown below. Splenocytes were identified based on FSC SSC parameters, excluding BHK-21 and RAW-264. Cells were then gated on CD19^+^ CD11b^−^ and GFP expression was monitored in HEL^+^ and HEL^−^ B cell. Data were obtained from two independent experiments with two splenocytes suspensions in each.

We co-cultured freshly isolated SW_HEL_ splenocytes with infected RAW-264 or BHK-21 cells, or exposed the splenocytes to free viruses (Figure 4). 48 h post co-culture, cells were harvested and GFP expression was monitored in both HEL^+^ and HEL^−^ B cells. Contrasting with our in vivo observations, we observed that both populations were equally sensitive to MuHV-4 infection when co-cultured with infected RAW-264, while they remained not infected with exposed to free virions or co-cultured with infected BHK-21 (Figure 4). Fitting with previous observations, percentages of infection were greater with a gp150-deficient virus. Overall, these in vitro data show that the absence of latently infected HEL^+^ B cells in vivo is not due to an intrinsic resistance of these cells to the virus.

### MuHV-4 induces transient B cell activation, but HEL^+^ B cells are excluded from late phases of the humoral response

**Figure 5:**
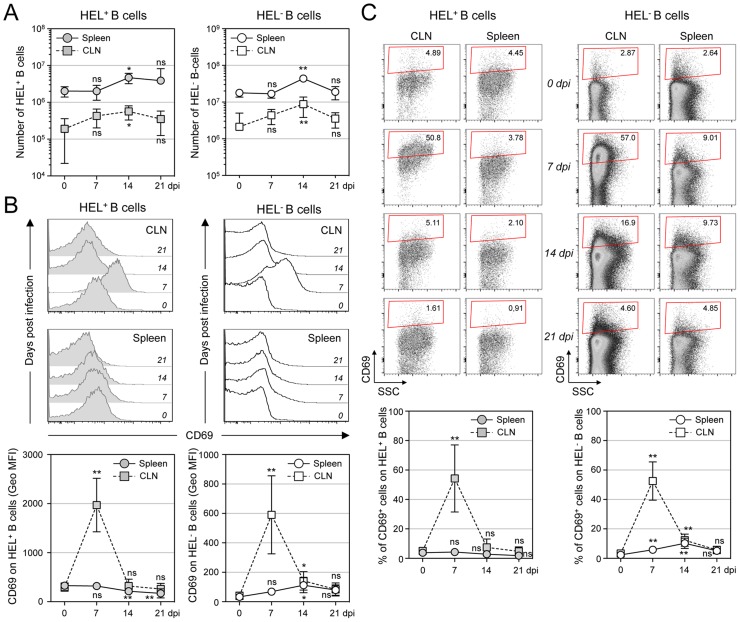
Proliferation and transient activation of total B cells. Spleens and CLN from YFP-MuHV-4 infected SW_HEL_ mice were harvested at 0, 7, 14 and 21 dpi and cells were stained with CD69 PE, CD19 APC-Cy7 and HEL-A647. (A) Live cells were enumerated, and the number of HEL^+^ and HEL^−^ B cells was established based on their frequency obtained from the FACS data. (B) Representative FACS histograms showing CD69 expression on HEL^+^ (grey curves) and HEL^−^ (white curves) B cells isolated from CLN and spleen at the different time points are shown. CD69 was monitored by measuring the Geo MFI and compiled values are shown in the graphic below. (C) On the same samples, the frequency of CD69^high^ cells was evaluated. Representative FACS plots are shown and compiled percentages are presented in the graphic below. These data were obtained from two independent experiments, with a total 5 to 6 mice per time point. In the graphics, mean values are reported and error bars represent the standard deviation.

MuHV-4 is known to induce proliferation of both T cells and B cells [30]. To evaluate the influence of MuHV-4 on early B cell activation, we performed a kinetic analysis monitoring the number and CD69 expression of HEL^+^ and HEL^−^ B cells isolated from spleen and cervical lymph nodes (CLN) (Figure 5). We observed an increased number of both HEL^+^ and HEL^−^ B cells in the spleen and CLN, which peaked at 14 dpi (Figure 5A), suggesting that MuHV-4-driven B cell proliferation does not rely on the infection status. We monitored CD69 expression by two complementary methods: measuring the intensity of CD69 expression (Figure 5B) and by evaluating the percentage of CD69^high^ cells (Figure 5C). In the spleen, beside a small population of CD69^high^ cells observed on HEL^−^ B cells at 7 and 14 dpi (Figure 5C), we did not detect a significant increase of CD69 expression on HEL^+^ and HEL^−^ B cells. On the opposite, in the CLN we observed a peak of CD69 expression on both HEL^+^ and HEL^−^ B cells at 7 dpi, which disappeared at 14 dpi. In both organs, YFP^+^ B cells were restricted to the HEL^−^ B cells indicating that this transient activation of HEL^+^ B cells is not sufficient to allow viral latency (Figure S2).

**Figure 6:**
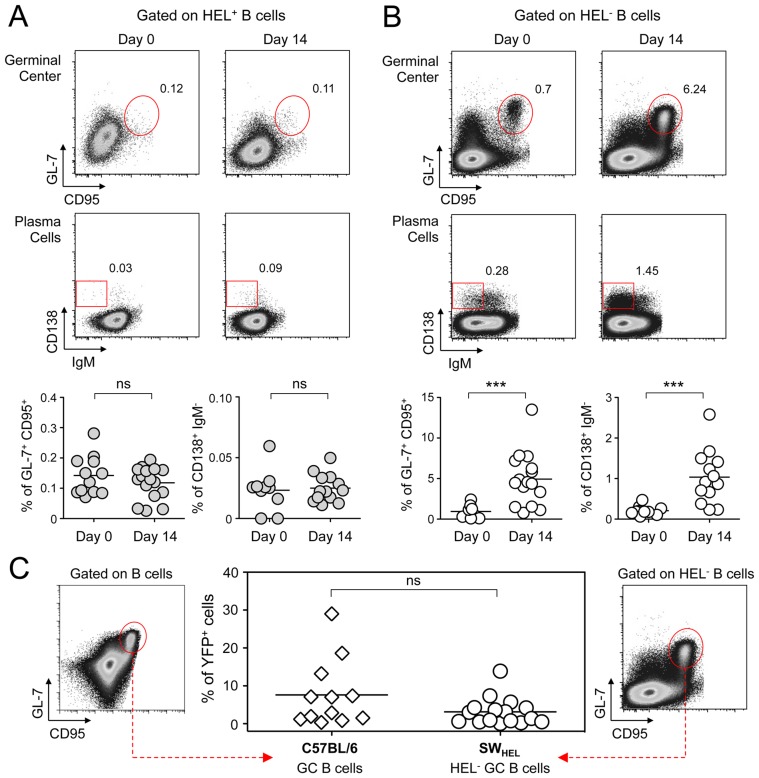
GC and plasma cell differentiation are restricted to the HEL^−^ B cells. Splenocytes were purified from SW_HEL_ mice at 0 and 14 dpi and analyzed by FACS. Cells were gated on (A) HEL^+^ or (B) HEL^−^ B cells for phenotype analysis, monitoring the frequency of GC (CD95^+^ GL-7^+^) and plasma cells (IgM^−^ CD138^+^). Top parts of the figures show representative FACS plots, bottom parts are the compilation of data obtained from different SW_HEL_ mice (GC: day 0 n = 12; day 14 n = 16/Plasma cells: day 0 n = 10; day 14 n = 13). (C) Germinal center cells in C57BL/6 B cells and HEL^−^ B cells harbor an equivalent frequency of YFP^+^ cells. Frequency of YFP^+^ cells in GC cells was evaluated 14 dpi by gating on the CD95^+^ GL-7^+^ population from total B cells of C57BL/6 mice (left dot plot and diamonds, n = 12) or from HEL^−^ B cells of SW_HEL_ mice (right dot plot and circles, n = 16). The bar represents the average percentage.

As MuHV-4 is known to establish latency in GC B cells, we next monitored the late phase of the B cell response by following the frequency of GC and plasma cells in HEL^+^ and HEL^−^ B cells (Figure 6A & 6B). Although our ELISPOT (Figure 1), proliferation (Figure 5A) and early activation (Figure 5B & 5C) data suggested a polyclonal B cell activation upon MuHV-4 infection, HEL^+^ B cells did not acquire a GC or plasma cells phenotype at 14 dpi (Figure 6A). In contrast, HEL^−^ B cells entered GC reactions and differentiated into plasma cells (Figure 6B). The frequency of YFP^+^ cells in HEL^−^ GC was ∼8%, in accordance to what we observed in C57BL/6 mice (Figure 6C).

**Figure 7:**
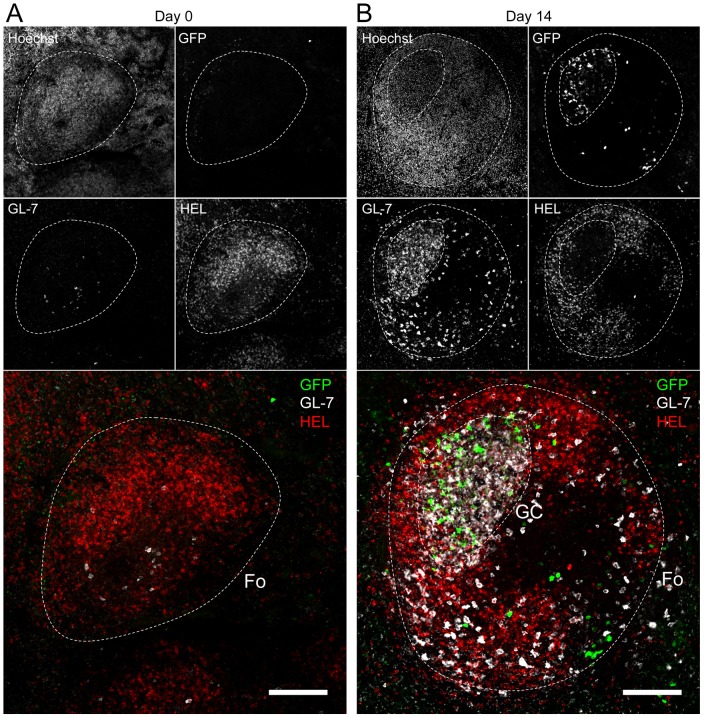
HEL^+^ B cells are excluded from the GC. Spleens were isolated from (A) day 0 (n = 2) and (B) day 14 (n = 4) infected SW_HEL_ mice for microscopy analysis. Spleens were treated as indicated in the material and methods, and stained with an anti-GFP, anti-GL-7, HEL and nuclei dye Hoechst. Separate channels are shown as thumbnails, in the merge image below nuclei were excluded for clarity. Follicles (Fo) were localized based on Hoechst signal, showing delimited clusters of nuclei. GCs were identified as clusters of GL-7^+^ cells. Spleen sections were thoroughly analyzed and images shown are typical organization of splenic follicles of SW_HEL_ mice at 0 and 14 dpi. At 14 dpi, infected cells were mainly localized within the GC, from which HEL^+^ B cells were systematically excluded. Scale bars represent 100 µm.

Spatial organization of the GC is an essential component of the B cell response as it dictates the interaction between B cells and the other cellular players such as follicular helper T cells and dendritic cells [31]. To have an insight into the organization of the HEL^+^ and HEL^−^ B cells in infected mice, we performed immunofluorescent staining on spleen sections from naïve and 14 dpi SW_HEL_ mice (Figure 7). As natural YFP signal was lost during fixation, infected cells were revealed with an Alexa-488 anti-GFP antibody. In naïve mice (Figure 7A), HEL^+^ B cells were homogeneously spread in the B cell area of the follicle, and no GFP^+^ or GC cells were observed. At 14 dpi, clusters of GL-7^+^ cells were present in the B cell area (Figure 7B), in which latently infected cells were found but HEL^+^ B cells were excluded. The number of GFP^+^ cells varied greatly between GCs; a heterogeneity also seen in C57BL/6 mice [32]. However, no matter the number of GFP^+^ cells present, we systematically observed an exclusion of the HEL^+^ B cells from the GC (Figure 7B).

These histological data confirm our phenotypical analysis (Figure 6) and overall these data show that while HEL^+^ B cells are sensitive to MuHV-4 infection in vitro (Figure 4) and get activated in vivo (Figure 5), they do not support latent infection and do not participate to the GC reaction induced by MuHV-4.

### SW_HEL_ mice develop an anti-MuHV-4 IgG response but fail to secrete long-lasting anti-HEL IgGs

**Figure 8:**
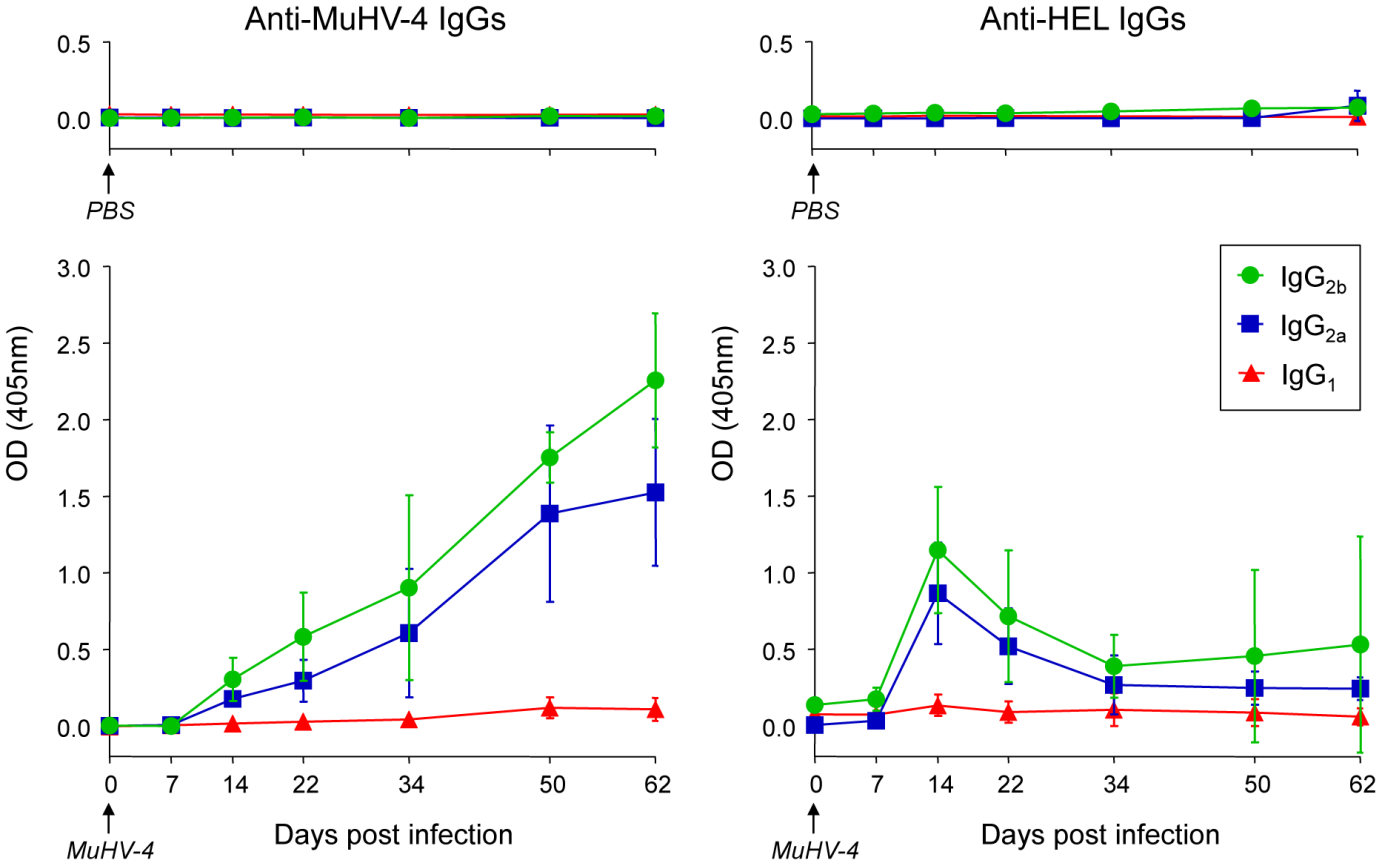
SW_HEL_ mice mount a long-lasting anti-MuHV-4 antibody response. SW_HEL_ mice were inoculated intranasally with PBS (n = 2) or MuHV-4 (n = 6). At indicated time points, mice were bled and sera were analyzed by ELISA to monitor levels of circulating anti-MuHV-4 and anti-HEL IgG_1_ (red triangles), IgG_2a_ (blue squares) and IgG_2b_ (green circles). For MuHV-4 infected mice, 3 independent experiments with 2 mice in each were analyzed. As no standard were available to quantify these antibodies, samples of different time points from identical mice were run together to be able to compare the absorbance at 405 nm.

To support our phenotypic and histologic observations, PBS or MuHV-4 challenged SW_HEL_ mice were bled to measure plasmatic levels of anti-MuHV4 and anti-HEL IgG_1_, IgG_2a_ and IgG_2b_ (Figure 8). We were not able to quantify the amount of circulating antibodies as no standards were available, but the magnitude of these responses was assessed by systematically analyzing the time points from identical mice together, limiting the impact of technical variations. MuHV-4 has been previously shown to trigger an anti-viral response dominated by the production IgG_2a_ and IgG_2b_ [8] and our kinetic analysis followed the same pattern (Figure 8, left graphic). This response appeared between 7 and 14 dpi and gradually increased. For the anti-HEL response, although we did not detect a HEL^+^ GC response, we observed a peak of anti-HEL IgG_2a_ and IgG_2b_ antibodies 14 dpi, which declined quickly thereafter (Figure 8, right graphic). Naïve SW_HEL_ mice have a basal level anti-HEL IgGs [21] and the expansion and transient activation of HEL^+^ B cells observed in the CLN after MuHV-4 infection (Figure 5) could account for this burst of anti-HEL IgGs. However, the fact that this anti-HEL response is transient indicates the absence of a long-term anti-HEL response, fitting with our previous observations.

### MuHV-4 does not benefit from acute B cell responses to establish latency in non-virus-specific B cells

Our data show that HEL^+^ B cells are not latently infected and do not participate in the GC response induced by MuHV-4 while they are equally sensitive to infection in vitro. Moreover, our ELISPOT data show that viral latency is not restricted to virus-specific B cells, indicating that latency is established in B cells of other specificities. This set of observations leads us to propose that the establishment of latency is not a stochastic event and takes place in a restricted population of polyclonal B cells. This model implies that MuHV-4 does not overcome the stimulatory signals provided by the BCR stimulation and the cognate CD4 help, but manages to benefit from it in order to settle in long-lived memory B cells.

To test if MuHV-4 could benefit from acute CD4-dependent B cell responses, we stimulated a physiological number of adoptively transferred HEL^+^ B cells in C57BL/6 mice with sheep red blood cells (SRBC) conjugated to recombinant HEL [23]. We used an adoptive transfer assay in order to avoid competition between HEL^+^ B cells in SW_HEL_ mice, in particular from being in too great an excess over the available SRBC-specific CD4 help. Indeed, SW_HEL_ immunized with SRBC-HEL showed a poor GC response (∼1% of GC HEL^+^ B cells, Figure S3A) when compared to C57BL/6 adoptively transferred with HEL^+^ B cells (∼70% of GC HEL^+^ B cells, Figure S3B). We controlled that SRBC-HEL could not induce an endogenous HEL-specific B cell response in C57BL/6 mice by co-transferring SRBC-HEL with or without HEL^+^ B cells and showed that transferred HEL^+^ B cells were required for the emergence of HEL^+^ GC B cells (Figure S3B).

**Figure 9:**
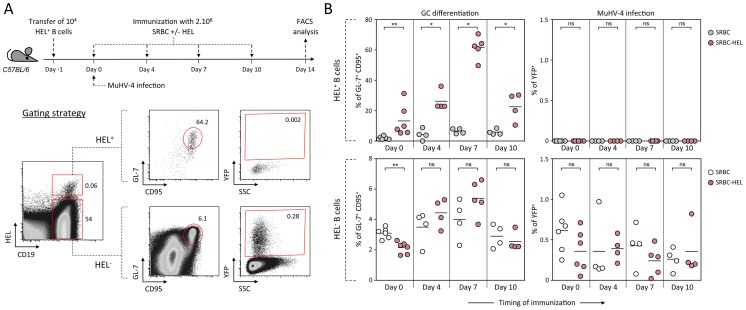
Activated HEL^+^ B cells remain refractory to MuHV-4 infection. (A) Experimental design: Bulk SW_HEL_ splenocytes containing 10^4^ HEL^+^ B cells were transferred in C57BL/6 mice 24 h prior YFP-MuHV-4 infection. Recipient mice were challenged intravenously by a single immunization with 2.10^8^ SRBC or SRBC-HEL performed at 0, 4, 7 or 10 dpi. At 14 dpi, spleens were collected and analyzed by FACS. As shown in the representative FACS plots, transferred HEL^+^ were identified with HEL-A647 staining while transferred HEL^−^ B cells could not be discriminated from endogenous B cells. On both HEL^+^ and HEL^−^ (transferred+endogenous) B cells, GC differentiation and percentage of infection was determined using GL-7/CD95 and YFP expression respectively. (B) For each time of immunization, frequency of GC B cells and YFP^+^ B cells were compared between SRBC and SRBC-HEL immunized mice in both HEL^+^ (top panel) and HEL^−^ B cells (bottom panel). Each dot represents an individual mouse and bars represent the average percentages. The data were obtained from 4 to 6 mice per experimental group.

As schematized in Figure 9A, we transferred HEL^+^ B cells 24 h before infection and immunized the infected mice with SRBC+/−HEL at 0, 4, 7 or 10 dpi. We decided to test different time of immunization, as it is currently unknown when the virus/B cell encounter happens and whether it infects naïve or activated B cells in vivo. At 14 dpi, we monitored the GC differentiation and percentage of infection in HEL^+^ and HEL^−^ B cells (Figure 9B). Immunization with SRBC-HEL at either of the time point tested triggered the GC differentiation of HEL^+^ B cells when compared to mice immunized with SRBC alone (Figure 9B, top left) while it did not affect the GC phenotype of HEL^−^ B cells (Figure 9B, bottom left). The magnitude of the GC response observed in HEL^+^ B cells was different between the time of immunization, certainly due to a mixed influence of the GC dynamic and survival of the transferred cells. While we could detect for the first time a HEL^+^ GC response in the context of MuHV-4 infection, these cells remained YFP^−^ (Figure 9B, top right), YFP^+^ cells being restricted to the HEL^−^ population (Figure 9B, bottom right), in which frequency of infection was not affected by SRBC-HEL immunization. We verified that adoptively transferred B cells could get latently infected by transferring WT CD45.1^+^ splenocytes into WT CD45.2^+^ recipient mice and showed that frequency of infection and GC differentiation was equivalent between donor and recipient cells (Figure S4).

These data support the fact that HEL^+^ GC B cells are resistant to MuHV-4 latency and that MuHV-4 does not benefit from acute B cell responses to establish latency in non-virus-specific B cells, likely relying on other mechanisms yet to be identified.

## Discussion

In this study, we attempted at better understanding how γ-herpesviruses establish latency in B cells with a particular focus on the role of the BCR specificity. By following HEL^+^ B cells in MuHV-4 infected SW_HEL_ mice, we were able to monitor the behavior of non-virus specific B cells during the establishment of MuHV-4 latency and showed that those cells were excluded from the latently infected population.

Previous studies have established that MuHV-4 latency depends on B cell activation and proliferation [33], but it is still not clear whether MuHV-4 can drive such activation independently of BCR specificity. When we compared the proliferation and CD69 expression of HEL^+^ and HEL^−^ B cells, we observed that both populations behave in a similar manner, with proliferation in both spleen and CLN and a transient CD69 upregulation in the CLN. This confirms previous work that showed CD69 upregulation on B cells exposed to MuHV-4 in vitro and a temporary B cell proliferation in MHC-II-deficient I-A^b^−/− mice [7]. However, HEL^+^ B cells did not participate to the long-term humoral response, as they did not differentiate into GC or plasma cells. We think we observed here two distinct waves of activatory signals. The first wave triggering a non-specific activation of the global B cell population, followed by a second wave that promotes the differentiation into GC of a restricted pool of B cells. The respective role of these two waves in the establishment of latency is not completely clear, but the first wave of activation is not sufficient to allow the establishment of latency in B cells, as supported by the fact that HEL^+^ B cells do not get latently infected. While we do not identify the mechanism driving the first wave of activation, it has been shown that both T cells and B cells are responsible for the MuHV-4 driven splenomegaly [30], suggesting that the first wave of activation is not due to factors specific of the B cell response, and is probably cytokine mediated.

**Figure 10:**
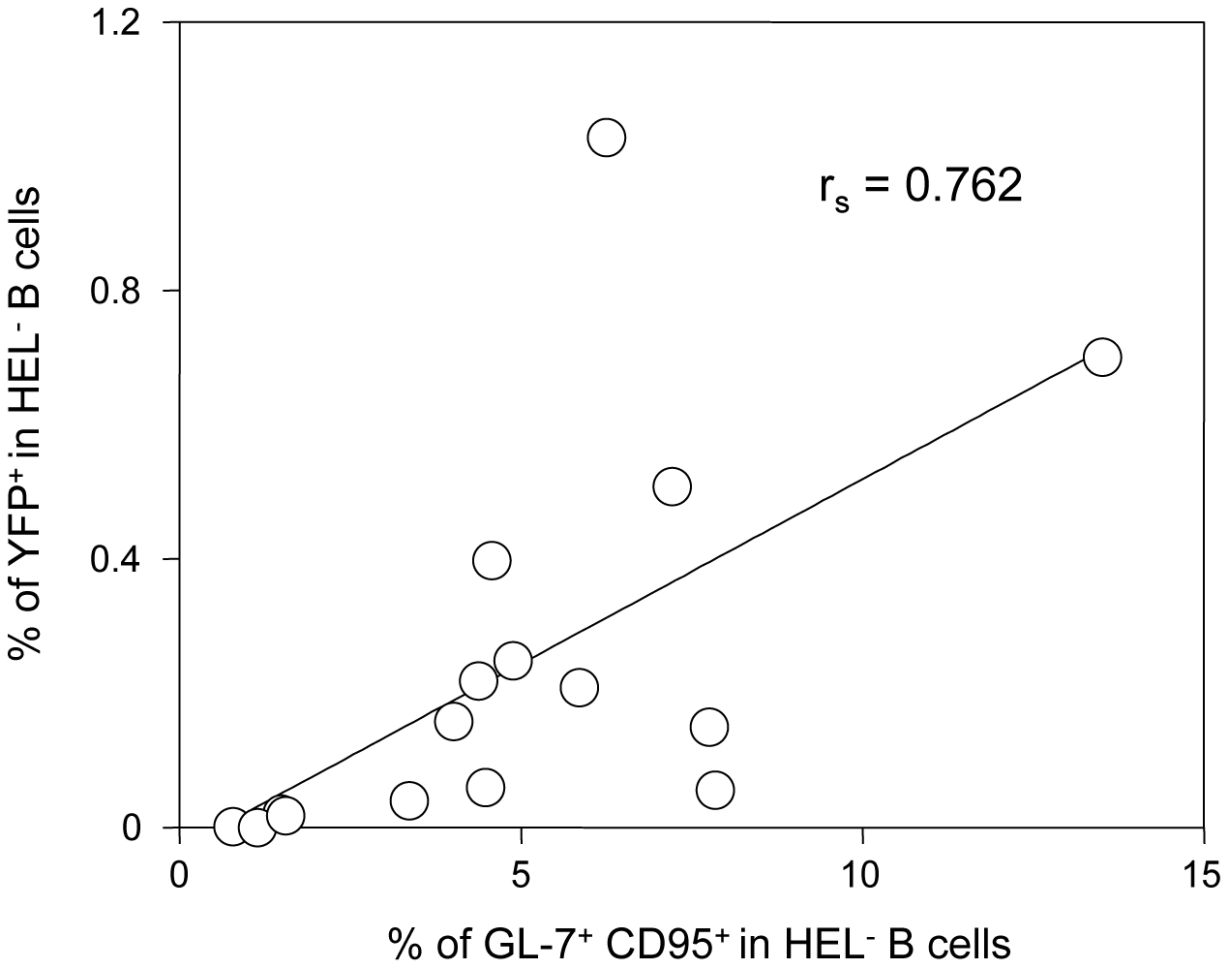
The frequency of infection correlates with the magnitude of the germinal center response in HEL^−^ B cells. To study the interrelationship between the GC response and the frequency of YFP^+^ cells, matched data obtained from SW_HEL_ mice 14 dpi (n = 16) were plotted against each other. For each SW_HEL_ mouse, the Y-axis shows the frequency of YFP^+^ cells in HEL^−^ B cells (data from Figure 2A) and the X-axis shows the frequency of GC (data from Figure 6B). R_s_ was calculated using Spearman's rank correlation coefficient.

Concerning the second wave of activation, correlation analysis performed on our dataset showed that frequency of YFP^+^ cells in HEL^−^ B cells correlates positively with the magnitude of the GC response (Figure 10). This is in accordance with recent observations by Collins et al who observed a positive correlation between the frequency of YFP^+^ cells and the frequency of follicular helper T cells, an essential player of the GC response [10]. That said, the fact that HEL^+^ B cells are sensitive to the virus in vitro but do not get latently infected and are excluded from the GC reaction go against a model where MuHV-4 could drive a stochastic manipulation of the B cells and would instead rely on BCR specificity.

One previous study looked at the influence of MuHV-4 on non-virus specific B cells by reconstituting μMT B cell −/− mice with B cells from MD4 mice (designated HELMET mice [34]). In MuHV-4 infected HELMET, HEL^+^ B cells expressed CD69 and proliferated but contrary to our results, MuHV-4 latency was detected in HEL^+^ B cells by PCR. The absence of competition in HELMET mice, where B cells are all HEL-specific could account for these discrepancies. Indeed, in SW_HEL_ mice, HEL^+^ B cells coexist with a majority of polyclonal B cells. Our in vitro infection by co-culture assay supports the fact that HEL^+^ B cells are sensitive to MuHV-4, suggesting that a selection mechanism might occur in vivo, leading to the disappearance of these infected cells. In HELMET mice, the absence of competition could allow for the survival of latently infected HEL^+^ B cells. This role for competition is supported by the work of Kim et al [35] who studied how latency evolved in mice containing CD40^+^ and CD40^−^ B cells. Although the two populations got latently infected, latency was ultimately lost in CD40^−^ B cells and GC differentiation was restricted to CD40^+^ B cells.

To explore whether the presence of CD4 T cell dependent antigens could be involved in the selection of latently infected cells, we triggered an endogenous anti-HEL response concomitantly with MuHV-4 infection. Although we were able to induce the emergence of HEL^+^ GC B cells, these cells remained refractory to latent infection. These data support that, at least in our experimental setting, MuHV-4 cannot hitchhike an acute humoral response to gain access to GC B cells and might instead benefit from other antigen/BCR interactions. In humans, it is estimated that up to 20% of a normal B cell repertoire is made of self-reactive B cells that need to be constantly kept under control [36], [37]. One of the tolerance mechanism is the induction of a functional unresponsive state known as anergy, which requires endogenous BCR signaling [38], [39]. The high prevalence of self-reactive B cells offers a good opportunity for γ-herpesviruses to manipulate these processes in order to retrieve B cells from their anergic state and promote polyclonal B cells activation in an antigen-dependent manner. Supporting this point, recent studies performed in humans [40] and mice [41], [42] have shown that γ-herpesviruses are found in self-reactive B cells. Two studies have explored MuHV-4 impact on anergic B cells [42], [43], but it is still not clear how MuHV-4 can modulate this processes. SW_HEL_×ML5 mice [21], in which HEL^+^ B cells are anergic due to the presence of soluble HEL, could be an alternative model to study the influence of the virus on competent anergic B cells.

Further work will be required to elucidate how MuHV-4 promotes GC B cells differentiation independently of their infection status, and how this event is linked to the establishment of latently infected B cells in memory B cells. γ-herpesviruses are known to be mildly pathogenic in immune-competent hosts and it has been shown that MuHV-4 latent infection does not induce autoimmune disorders and actually confers protection in lupus-prone animals [41]. This highlights the fact that the interrelationship between MuHV-4 and B cells is complex and that co-evolution between γ-herpesviruses and their host as allowed for the emergence of subtle mechanisms that promote B cell activation but limits associated immune disorders in order to establish life-long latency.

## Materials and Methods

### Ethics statement

This study was carried out in strict accordance with the recommendations of the Portuguese official Veterinary Directorate, which complies with the Portuguese Law (Portaria 1005/92). The Portuguese Experiments on Animal Act strictly comply with the European Guideline 86/609/EEC and follow the FELASA (Federation of European Laboratory Animal Science Associations) guidelines and recommendations concerning laboratory animal welfare. All animal experiments were approved by the Portuguese official veterinary department for welfare licensing under the protocol number AEC_2010_017_PS_Rdt_General and the IMM Animal Ethics Committee.

### Mice

SW_HEL_ mice [21] were obtained from Dr Antonio Freitas, Institut Pasteur, Paris, in accordance with Dr Robert Brink, Garvan Institute, Melbourne. To screen for expression of the V_H_10_tar_ heavy chain and the V_κ_10-κ light chain genotyping was performed on DNA isolated from mouse-tails using DirectPCR solution (Viagen). Mice heterozygous for both genes were used for experiments. HEL^+^ B cells were identified by FACS and confocal microscopy (see details below) by direct labeling with recombinant HEL (Sigma-Aldrich) conjugated with Alexa 647 (noted HEL-A647). This conjugation was made with the Alexa Fluor Antibody Labeling Kit (Invitrogen) following manufacturer instructions. A Bio-Gel P-6 (Biorad) loaded column was used to separate HEL-A647 conjugates from free dye. C57BL/6, CD45.1 and CD45.2 mice and were purchased from Charles Rivers Laboratories. Mice were between 7 and 15 weeks old at time of infection and were sacrificed by CO_2_ inhalation or cervical dislocation.

### Viruses

The YFP expressing MuHV-4 [4] was obtained from Dr Samuel Speck, Emory Vaccine Center, Atlanta. EF1α-eGFP^+^ MuHV-4 and EF1α-eGFP^+^-gp150^−^ MuHV-4 [29] were obtained from Philip Stevenson, University of Cambridge, Cambridge. Viral stocks were prepared by infecting BHK-21 cells and titrated by plaque assay using previously published procedures [44], [45]. For infections, mice were anaesthetized with isoflurane and inoculated intranasally with 10^4^ pfu of YFP-MuHV-4 under 20 µl of PBS.

### IgGs secreting cells ELISPOT assay

ELISPOT assay to enumerate MuHV-4 specific B cells was adapted from [8]. Briefly, purified MuHV-4 were disrupted for 10 min in PBS+0.05% Triton X-100 and plated at 5×10^6^ PFU/well in 96-well MultiScreen HA mixed cellulose filter plates (Millipore, Billerica, MA). Plates were incubated overnight at 4°C, washed with PBS and blocked for 1 h at 37°C with complete medium (RPMI-1640+10% heat inactivated FBS, 2 mM glutamine, 100 U/ml penicillin and streptomycin and 1 mM sodium pyruvate). Latently infected (CD19^+^ YFP^+^) and non-infected (CD19^+^ YFP^−^) B cells were sorted from spleens on a FACS Aria (BD Biosciences). Serial four-fold dilutions of sorted cells were prepared in complete medium and added under 100 ul/well in four replicate wells per cell amount. Cells were incubated overnight at 37°C in a humid 5% CO_2_ incubator. Plates were washed with PBS and incubated for 2 h at room-temperature with Alkaline phosphatase (AP)-conjugated rabbit anti-mouse IgG (H+L) antibodies (Southern Biotech) diluted 1/500 in PBS+0.5% FBS. After thorough washes spots were revealed at room temperature with 1 mg/ml of 5-bromo-4-chloro-3-indolyl phosphate (Sigma) in diethanolamine buffer. Upon optimal spot development plates were washed and dried. Blue spots representing single antibody-secreting cells (ASC) were counted under an Olympus SZ51 microscope. Total number of ASC were determined as described above except that plates were coated with 0.5 µg/well of a goat anti-mouse κ antibodies (Southern Biotech) diluted in PBS.

### In vitro infection by co-culture assay

In vitro infection by co-culture assay was adapted from [29]. 24 h prior co-culture, 3.10^5^ RAW-264 and BHK-21 cells were seeded in 24-well plate. After cell adhesion (4–6 h), media was removed and cell infected overnight with indicated MuHV-4 at 9.10^5^ pfu per condition. The next day, spleens were harvested and single cell suspensions were prepared by spleen disruption, filtration on 100 µm cell strainer and red blood cells removal by centrifugation on ficoll gradient (Biowest). Splenocytes were washed and added to cells at 10^6^/well. As a negative control, splenocytes were exposed to free viruses. In order to have enough cells to work with, spleens from two SW_HEL_ mice were pooled. For each experiments, two suspensions were analysed in parallel. After 48 h of co-culture, cells were harvested and stained with CD11b (to exclude RAW-264 cells), CD19 and HEL. Infection of RAW-264 was systematically evaluated 24 h post infection by monitoring GFP expression in cells not co-cultured with splenocytes (data not shown).

### Flow cytometry

Single cell suspensions were prepared from spleens. Red blood cells were lysed in hypotonic NH_4_Cl and stainings were performed at 4°C in PBS+4% FCS and 1 mM EDTA. Briefly, cells were blocked by 10 min incubation with FcBlock (anti-CD16/32, 2.4G2, BD bioscience), washed, and stained for 20 min. For biotinilated antibodies, extra 20 min incubation with streptavidin was performed. MuHV-4 infected cells were monitored based on their endogenous YFP expression. The following antibodies were used: anti-CD69 PE (H1.2F3), anti-CD95 PE (Jo2), anti-CD19 APC-Cy7 or APC-H7 (1D3), anti-IgM PE (R6-60.2), anti-IgD Biotin (11-26c.2a), CD11b PE or v450 (M1/70) (BD Biosciences); CD45.2 Brilliant Violet 510 (104) (BioLegend); anti-CD45.1 PeCy7 (A20), and anti-GL-7 Biotin (Ebioscience). Streptavidin-Cy5 (BD Biosciences) was used to reveal biotinilated antibodies. HEL specific B cells were identified with the HEL-A647 conjugate described above. Samples were acquired on a FACS Canto or on a LSR Fortessa (BD Biosciences), using DIVA software (BD Biosciences) for acquisition and Flowjo v.6.4.7 (Tree Star) for analysis. Cells were gated on live cells based on FSC/SSC parameters and cell doublets were excluded based on FSC-W signal.

### Immunofluorescence histology

Spleens were fixed overnight at 4°C in periodate-lysin-paraformaldehyde (PLP) [46], [47] and dehydrated by successive 2 h incubation in 10%, 20% and 30% sucrose solutions at 4°C. Spleens were then embedded in OCT (Tissue Tek), frozen and sectioned (40 µm). For immunofluorescence staining, sections were encircled with a Fatpen and rehydrated 10 min in phosphate buffer. All incubations were made in humid chamber, protected from light. Sections were permeabilized for 1 h at room temperature in 1% triton and blocked 1 h in 1% BSA+FcBlock (anti-CD16/32, 2.4G2, BD bioscience). Stainings were performed using the following reagents: anti-GFP Alexa 488 (Invitrogen), anti-GL-7 Biotin (GL-7, Ebioscience), HEL-A647 (described above) and Hoechst 33343 (Invitrogen). Streptavidin Alexa568 (Invitrogen) was used for biotinilated antibodies, incubated 1 h at room temperature. Slides were washed, mounted in Fluoromount-G (SouthernBiotech) and kept at 4°C. Images were acquired on a LSM 510 META point scanning confocal microscopes (Zeiss) and analyzed using LSM Image Browser (Zeiss) and Photoshop CS2 (Adobe).

### Cell sorting and in vitro reactivation assay

HEL^+^ B cells (CD19^+^, HEL^+^) and HEL^−^ B cells (CD19^+^, HEL^−^) were sorted using a FACS Aria (BD Biosciences) and used for in vitro reactivation assay to quantify latent infection. Serial dilutions of freshly isolated cells were co-cultured with BHK-21 in complete media supplemented with 50 µg/ml of Gentamycin (Invitrogen). Lysing half of the sorted cells by a quick freeze/thaw cycle before coculture allowed us to assess the presence of preformed viral particles, indicative of lytic infection. After 5 days, BHK-21 were fixed with 4% paraformaldehyde and stained with toluidine blue for plaque counting. The number of plaques in each sample was expressed as plaques forming unit (pfu)/10^7^ cells.

### Limiting dilution PCR analysis

The frequency of virus-genome-positive cells was determined from pools of 2 to 3 spleens by limiting dilution combined with real-time PCR as previously described [48]. Sorted HEL^+^ and HEL^−^ B cells were serially two-fold diluted and eight replicates of each dilution were analysed by real time PCR (Rotor Gene 6000, Corbett Life Science). The primer/probe sets were specific for the MuHV-4 M9 gene (5′ primer: GCCACGGTGGCCCTCTA; 3′ primer: CAGGCCTCCCTCCCTTTG; probe: 6-*FAM*-CTTCTGTTGATCTTCC–*MGB*). Samples were subjected to a melting step of 95°C for 10 min followed by 40 cycles of 15 s at 95°C and 1 min at 60°C. Positive *vs.* negative reactions were scored using the Rotor Gene 6000 software. Our data were compatible with the single-hit Poisson model (SHPM) as tested by modeling the limiting dilution data according to a generalized linear log-log model fitting the SHPM and checking this model by an appropriate slope test as described [3], [49]. A regression plot of input cell number against log fraction-negative samples was used to estimate the frequency of cells with viral genomes. Estimation of the cell subset frequency of MuHV-4 infection consisted of computation by maximal-likelihood estimation as follows: let *f* be the estimate of the cell frequency; the maximum likelihood of *f* is the value of *f* that maximizes
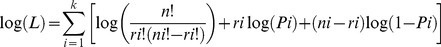
where log(*L*) is the natural logarithm of the likelihood function *L* and *Pi* is given by *Pi* = *exp*(-*f xi*) according to the SHPM. The variance of *f* was calculated as the negative reciprocal of the second derivative of log(*L*), var(*f*) = 1/[*d* ^2^ log(*L*)/*df* ^2^]. The 95% confidence interval (CI) for *f* was calculated as 95% CI (*f*) = *f*±1.96SE (*f*). Abbreviations are as follows: *k* = the number of groups of replicate PCRs, numbered *i* = 1, 2, … *k*; *ni* = the number of replicate reactions; *ri* = the number of observed negative PCRs; and *mi* = the observed fraction of negatives (*mi* = *ri/ni*).

### Adoptive transfers and SRBC+/−HEL immunization

Freshly isolated bulk splenocytes from SW_HEL_ mice containing 10^4^ HEL^+^ B cells were transferred into C57BL/6 mice by intravenous injection, as previously described [50]. Sheep red blood cells (SRBC) were obtained from Miguel Fevereiro, Laboratório Nacional de Investigação Veterinária, Lisbon. Recombinant HEL was covalently conjugated to SRBC with 1-ethyl-3-(3-dimethylaminopropyl)-carbodiimide hydrochloride (Sigma-Aldrich) as described [25]. Conjugation was confirmed by FACS by staining mock or HEL-conjugated SRBC with the HyHEL10 (an anti-HEL IgG_1_ [21]) followed by an anti-IgG_1_ APC (BD Biosciences). HEL-A647 was used to identify transferred HEL^+^ B cells.

### Anti-HEL and anti-MuHV-4 IgGs ELISA

To measure anti-HEL and anti-MuHV-4 antibody production, sera were regularly collected by facial-vein bleeding. To measure HEL-specific antibodies, maxisorp plates (Nunc) were coated with 60 µl of recombinant HEL (10 µg/ml) diluted in NPP buffer (adapted from [21]). For MuHV-4 antibodies, plates were coated with viral particles disrupted with 0,1% triton and diluted in NPP buffer (adapted from [7]). Coated plates were incubated overnight at 4°C and blocked for 1 h with 100 µl PBS+1% BSA. Sera were diluted to 1/200 in PBS+0,1% BSA and 50 µl were incubated 2 h at room temperature. IgG_1_, IgG_2a_ and IgG_2b_ subclasses were measured using 50 µl of anti-mouse IgG_1_, IgG_2a_ and IgG_2b_ conjugated to alkalyne-phosphatase (SouthernBiotech) diluted to 1/500 and incubated 1 h at room temperature. Bound antibodies were revealed using 100 µl of 1 mg/ml P-Nitrophenyl Phosphate (MP Biomedical) prepared in NPP buffer and incubated 40 min at 37°C. Absorbance was measured at 405 nm.

### Statistics

p values were calculated using non-parametric Mann-Whitney U test; ns indicates p>0.05, * indicates p≤0,05, ** indicates p≤0,005, and *** indicates p≤0,001.

## Supporting Information

Figure S1 **B-2 lineage represents the majority of latently infected B cells.** C57BL/6 (n = 5) were infected with YFP-MuHV-4 and spleens were analyzed 14 dpi. Cells were stained with CD19, CD5 and CD43 to identify B-2 (CD5^−^ CD43^−^), B-1a (CD5^+^ CD43^+^) and B-1b (CD5^−^ CD43^+^) B cells. (A) Representative FACS plots from YFP^−^ (left) and YFP^+^ (right) B cells are shown. (B) Average population percentages obtained from the 5 mice are shown for the YFP^−^ (grey bars) and YFP^+^ (black bars) B cells.
(TIF)

Figure S2 **MuHV-4 is restricted to HEL^−^ B cells in both LNs and spleen (complement to** **Figure 5** **).** Spleens and CLN from YFP-MuHV-4 infected SW_HEL_ mice were harvested at 0, 7, 14 and 21 dpi and cells were stained with CD69 PE, CD19 APC-Cy7 and HEL-A647. Frequency of infected cells was monitored in HEL^+^ (Left panel) and HEL^−^ B cells (right panel) based on YFP expression. Representative FACS plots are shown and compiled percentages are presented in the graphic below. These data were obtained from two independent experiments, with a total of 5 to 6 mice per time point. In the graphics, mean values are reported and error bars represent the standard deviation.
(TIF)

Figure S3 **Poor GC response in SW_HEL_ mice and absence of endogenous HEL^+^ B cell activation in C57BL/6 challenged with SRBC-HEL.** (A) SW_HEL_ mice were immunized intravenously with 2.10^8^ SRBC (n = 3) or 2.10^8^ SRBC-HEL (n = 3). 7 days post-challenge splenocytes were harvested and analyzed by FACS. Representative FACS plots shows frequency of GC cells (CD95^+^ GL-7^+^) in HEL^+^ B cell from mice challenged with SRBC or SRBC-HEL. (B) C57BL/6 were immunized intravenously with 2.10^8^ SRBC-HEL in presence (n = 3) or absence (n = 3) of co-transferred 10^4^ HEL^+^ B-cells. 7 days post-challenge splenocytes were harvested and analyzed by FACS. Representative FACS plots shows the frequency of HEL^+^ B-cells and their GC phenotype (CD95^+^ GL-7^+^) in each condition. A HEL^+^ B cell population with a GC phenotype was only detected when HEL^+^ B cells were co-transferred with SRBC-HEL, indicating that SRBC-HEL alone induced an undetectable HEL-specific response in C57BL/6.
(TIF)

Figure S4 **Adoptively transferred B cells get latently infected.** 24 h prior MuHV-4 YFP infection, CD45.2 C57BL/6 recipient mice (n = 6) received intravenously 10^7^ bulk splenocytes freshly isolated from CD45.1 C57BL/6 donor mice. At 14 dpi, spleens were isolated and cells stained with anti-CD19, CD95 and GL-7 as well as with anti-CD45.1 and CD45.2 in order to discriminate between donor (CD45.1^+^) and endogenous (CD45.2^+^) B cells. MuHV-4 infection in CD45.1^+^ and CD45.2^+^ B cells was evaluated by monitoring the frequency of YFP^+^ cells in each population (top panel). GC phenotype was assessed by monitoring CD95 and GL-7 expression on CD45.1^+^ and CD45.2^+^ B cells (central panel) as well as on YFP^+^ B cells in each population (bottom panel). For each panel, representative FACS plots and compiled data are shown. Bars represent average percentages.
(TIF)
