# Supplementary figures and images for: Establishment of Murine Gammaherpesvirus Latency in B Cells Is Not a Stochastic Event
Source: PLoS Pathog. 2014 Jul 31;10(7):e1004269. doi: 10.1371/journal.ppat.1004269 (PMC4117635; doi:10.1371/journal.ppat.1004269)

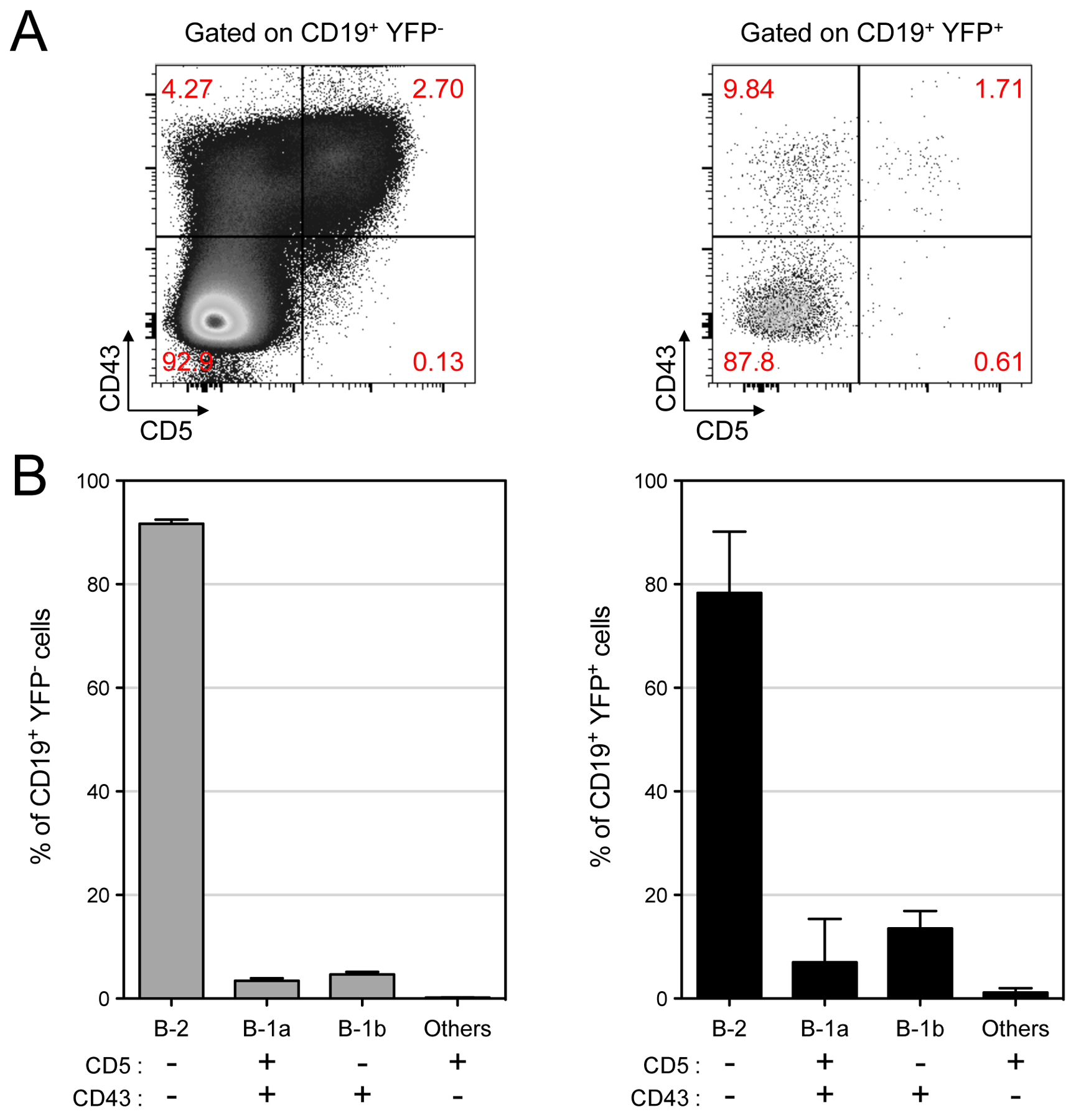

Supplement: Figure S1 — B-2 lineage represents the majority of latently infected B cells. C57BL/6 (n = 5) were infected with YFP-MuHV-4 and spleens were analyzed 14 dpi. Cells were stained with CD19, CD5 and CD43 to identify B-2 (CD5− CD43−), B-1a (CD5+ CD43+) and B-1b (CD5− CD43+) B cells. (A) Representative FACS plots from YFP− (left) and YFP+ (right) B cells are shown. (B) Average population percentages obtained from the 5 mice are shown for the YFP− (grey bars) and YFP+ (black bars) B cells. (TIF) [file ppat.1004269.s001.tif]

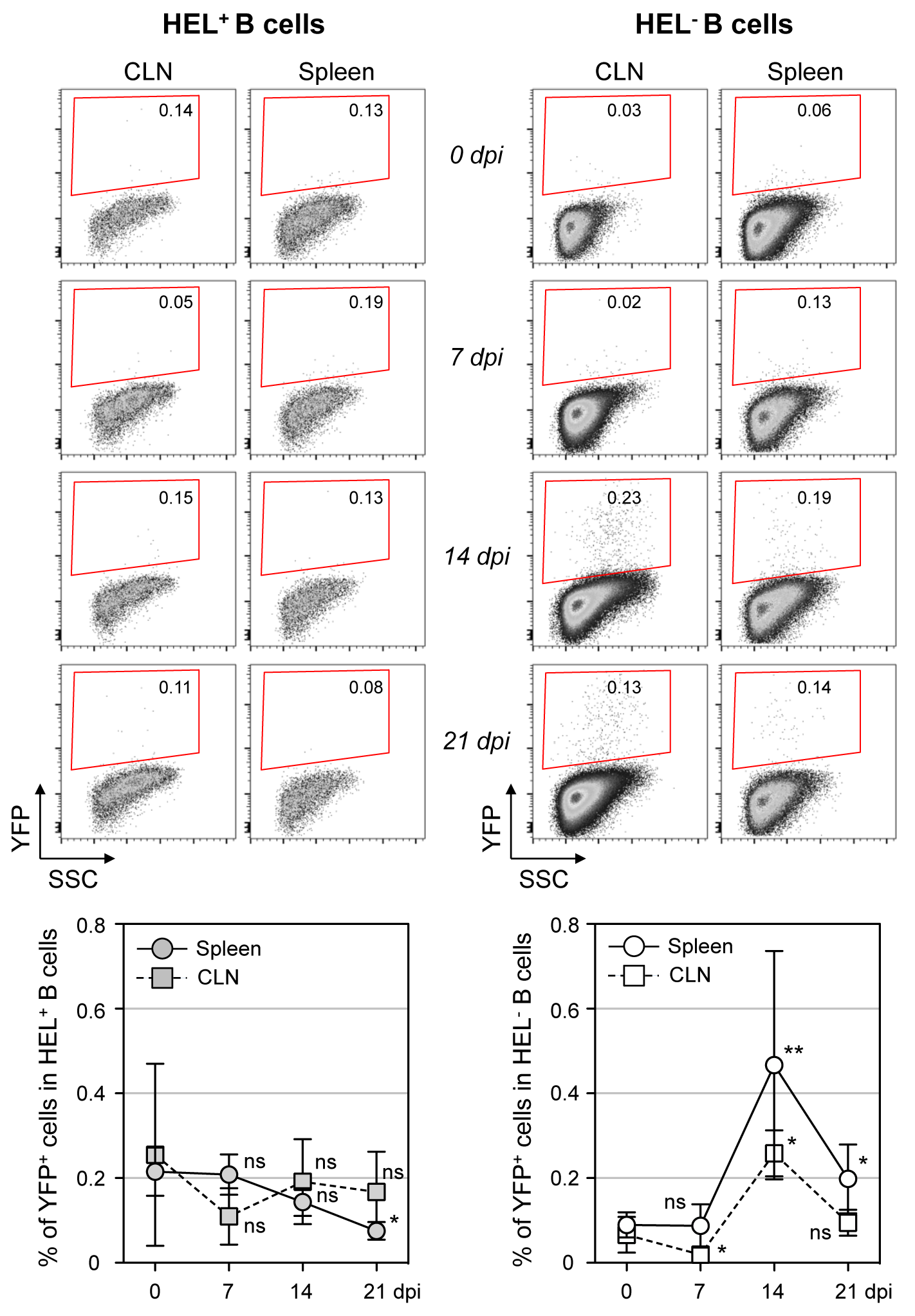

Supplement: Figure S2 — MuHV-4 is restricted to HEL− B cells in both LNs and spleen (complement to Figure 5 ). Spleens and CLN from YFP-MuHV-4 infected SWHEL mice were harvested at 0, 7, 14 and 21 dpi and cells were stained with CD69 PE, CD19 APC-Cy7 and HEL-A647. Frequency of infected cells was monitored in HEL+ (Left panel) and HEL− B cells (right panel) based on YFP expression. Representative FACS plots are shown and compiled percentages are presented in the graphic below. These data were obtained from two independent experiments, with a total of 5 to 6 mice per time point. In the graphics, mean values are reported and error bars represent the standard deviation. (TIF) [file ppat.1004269.s002.tif]

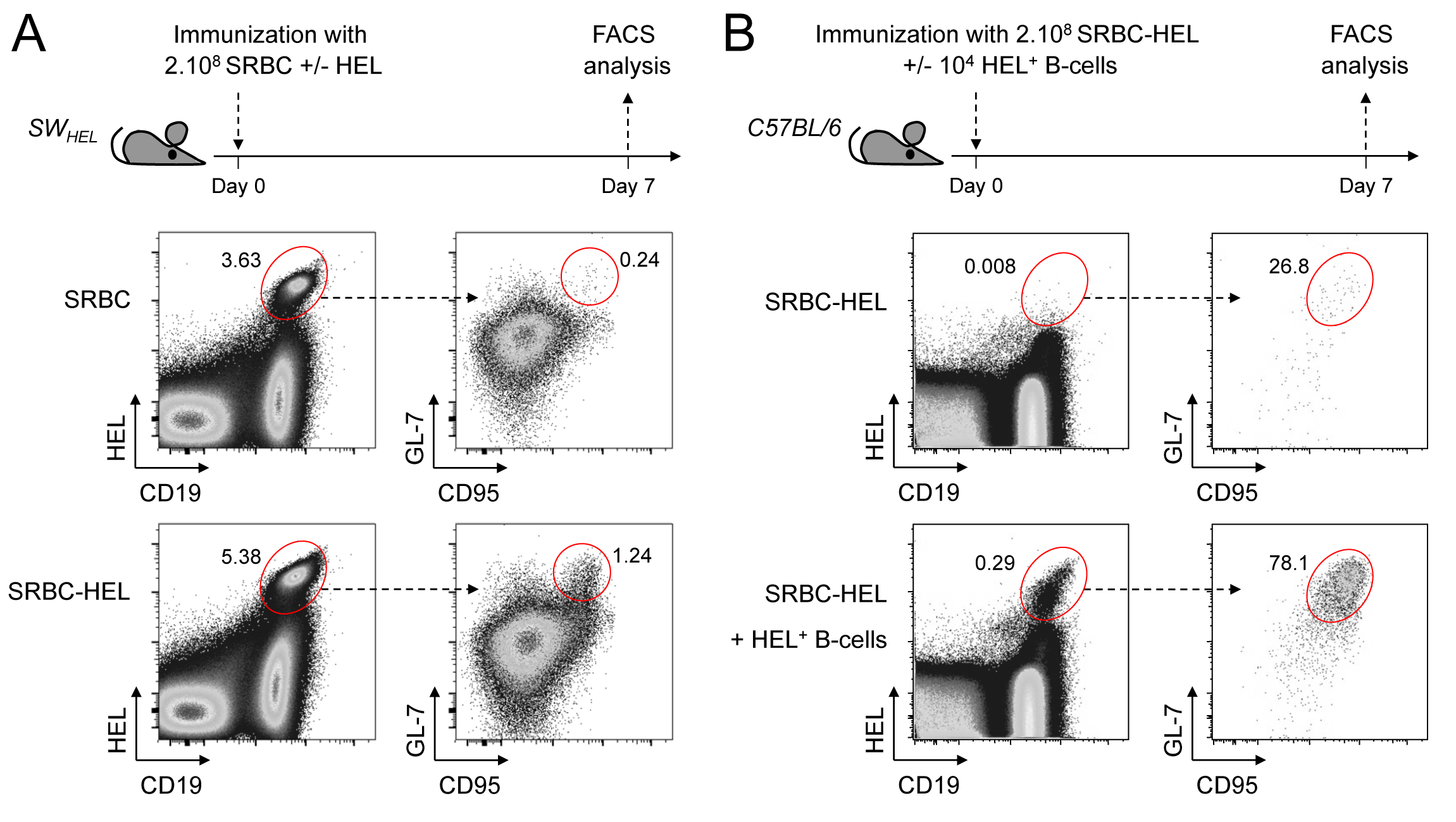

Supplement: Figure S3 — Poor GC response in SWHEL mice and absence of endogenous HEL+ B cell activation in C57BL/6 challenged with SRBC-HEL. (A) SWHEL mice were immunized intravenously with 2.108 SRBC (n = 3) or 2.108 SRBC-HEL (n = 3). 7 days post-challenge splenocytes were harvested and analyzed by FACS. Representative FACS plots shows frequency of GC cells (CD95+ GL-7+) in HEL+ B cell from mice challenged with SRBC or SRBC-HEL. (B) C57BL/6 were immunized intravenously with 2.108 SRBC-HEL in presence (n = 3) or absence (n = 3) of co-transferred 104 HEL+ B-cells. 7 days post-challenge splenocytes were harvested and analyzed by FACS. Representative FACS plots shows the frequency of HEL+ B-cells and their GC phenotype (CD95+ GL-7+) in each condition. A HEL+ B cell population with a GC phenotype was only detected when HEL+ B cells were co-transferred with SRBC-HEL, indicating that SRBC-HEL alone induced an undetectable HEL-specific response in C57BL/6. (TIF) [file ppat.1004269.s003.tif]

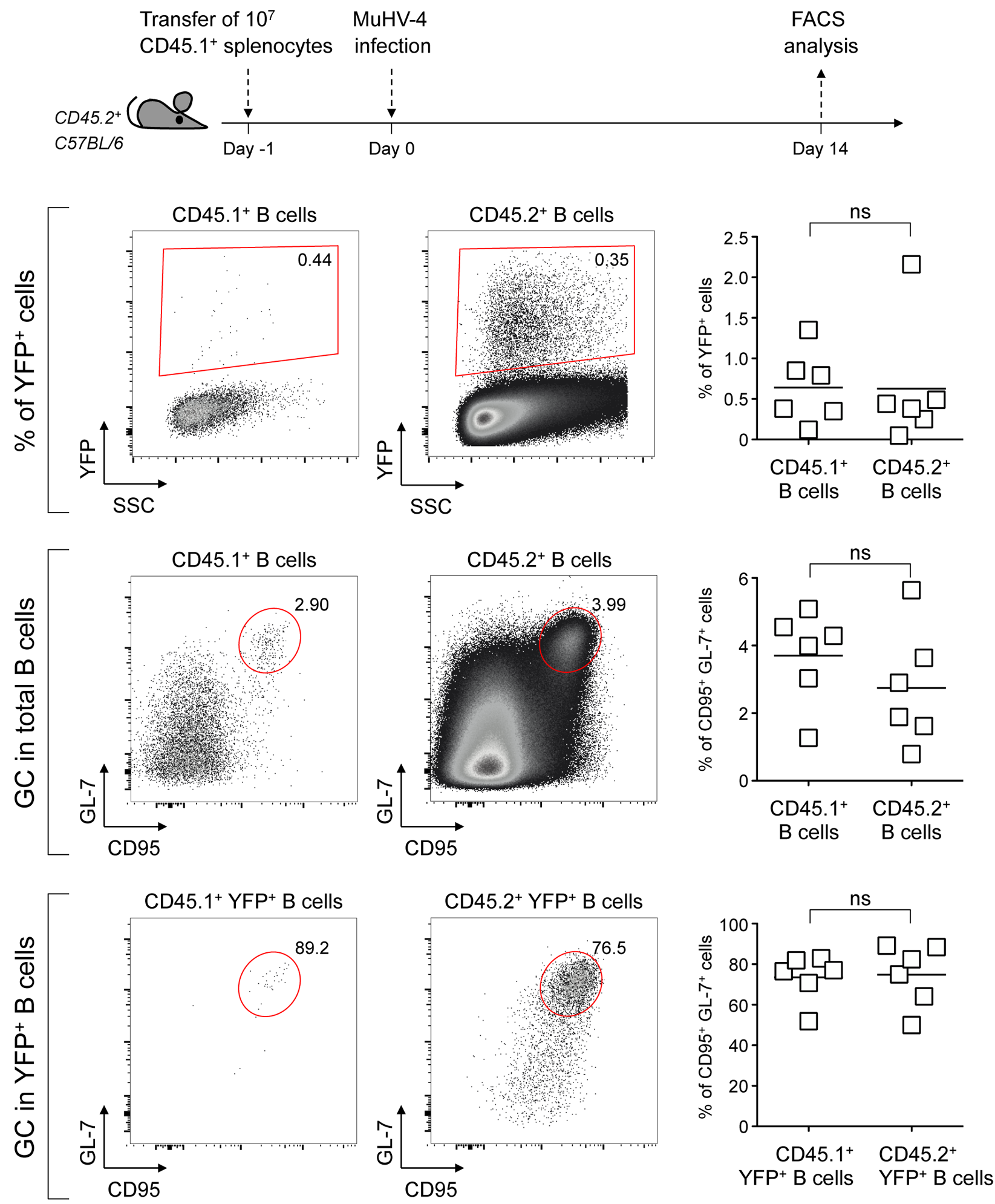

Supplement: Figure S4 — Adoptively transferred B cells get latently infected. 24 h prior MuHV-4 YFP infection, CD45.2 C57BL/6 recipient mice (n = 6) received intravenously 107 bulk splenocytes freshly isolated from CD45.1 C57BL/6 donor mice. At 14 dpi, spleens were isolated and cells stained with anti-CD19, CD95 and GL-7 as well as with anti-CD45.1 and CD45.2 in order to discriminate between donor (CD45.1+) and endogenous (CD45.2+) B cells. MuHV-4 infection in CD45.1+ and CD45.2+ B cells was evaluated by monitoring the frequency of YFP+ cells in each population (top panel). GC phenotype was assessed by monitoring CD95 and GL-7 expression on CD45.1+ and CD45.2+ B cells (central panel) as well as on YFP+ B cells in each population (bottom panel). For each panel, representative FACS plots and compiled data are shown. Bars represent average percentages. (TIF) [file ppat.1004269.s004.tif]
